# 

**Published:** 1895-11-16

**Authors:** 


### CONTENTS.

**A Stage in the Evolution of Medicine** - - - - 109
**Hunger as a Test of Poverty** - - - - - - 110
**Winter Quarters** - - - - - - - - - - - 110
**The Diagnosis of Aneurism.** By A. Pearce Gould, F.R.C.S. 111
**A Forgotten Medical Hero** - - - - - - - - 112
**Annotations.**—A Mortal Calling—The Dangers of Adulterated Boots—Our Homeless Ones - - - - - - - - 113
**Notes and News** - - - - - - - - - - - 114
**Scraps and Gleanings** - - - - - - - - - 123
**Medical Progress and Hospital Clinics**—
Ischio-Rectal Abscess and Fistula. By E. Percy Paton, M.D. 115
Progress in Surgery.—Genito-Urinary Surgery - - - 116
Progress in Otology - - - - - - - - - 119
New Appliances and Things Medical - - - - - 120
**The Institutional Workshop**—
Hospital Expenditure. — The Commissariat. VI. — The Resident Medical Officers - - - - - - - - - 121
Metropolitan Hospital Sunday Fund - - - - - 122

### EXTRA SUPPLEMENT.—THE NURSING MIRROR.

**News from the Nursing World.**—Our Princess—Notice to Policy Holders—Our Needlework Competition—Certificates of First Aid—Nursing at the Samaritan Hospital—East-end Mothers' Home—Training of Male Nurses—Prepaid Replies—The Personal Opinions of Attendants—Training at Glasgow Royal Infirmary—On and Off Duty—Unqualified to Advise—Short Items - - - - - - - - - - xlviii

**Elementary Physiology for Nurses.** By C. F. Marshall, late Surgical Registrar, Hospital for Sick Children, Great Ormond Street. XV.—The Sense Organs (*continued*) - - xlix

**Minor Appointments** - - - - - - - - - xlix

**On Urine Testing.**—III.—Sugar and Urea - - - - l

**London Nurses' Training Schools** - - - - - l

**Nursing in Florence.**—I. Ospedale di Santa Maria Nuova - li

**Our American Letter** - - - - - - - - li

**The Book World for Women and Nurses** - - - lii

**Everybody's Opinion** - - - - - - - - lii

**The Princess Maud Marriage Present Fund** - - liii

**Appointments** - - - - - - - - - - liii

**Notes and Queries** - - - - - - - - - liii

**Presentations** - - - - - - - - - - liii

**Royal British Nurses' Association** - - - - - liii

**A Coincidence** - - - - - - - - - - liv

**Novelties for Nurses** - - - - - - - - liv

